# The prevalence of the syndrome of inappropriate antidiuretic hormone secretion (SIADH) in brucellosis patients: Systematic review and meta-analysis

**DOI:** 10.1016/j.amsu.2022.103340

**Published:** 2022-02-05

**Authors:** Mouhand F.H. Mohamed, Ashraf O.E. Ahmed, Mhd Baraa Habib, Mohamed Abdalla, Yazan Salah Almohtasib, Hamad F.H. Mohamed, Zeinab.A.S. Mohamed, Lina Abdalla, Ahmed Osman Saleh

**Affiliations:** a Department of Medicine, Hamad Medical Corporation, Doha, Qatar; b Texas Vascular and Vein Center, Fort Worth, Texas, USA; c Endocrinology and Diabetes, HMC, Qatar

**Keywords:** SIADH, Brucellosis, Hyponatremia, Vasopressin, ADH

## Abstract

**Background:**

Hyponatremia is a prevalent electrolyte abnormality amongst hospitalized patients. The syndrome of inappropriate antidiuretic hormone secretion (SIADH) is a common cause of hyponatremia. Minimal literature described an association between SIADH and brucellosis. This paper aimed to systematically review the literature to synthesize the prevalence of SIADH in brucellosis patients.

**Methods:**

We comprehensively searched PubMed, EMBASE, and Google scholar for observational studies examining the prevalence of SIADH in brucellosis patients. There were no age, language, or date limitations. We used a prevalence meta-analysis using the random-effects model with double arcsine and back transformation. I squared (I2) was used to determine heterogeneity. The MetaXl software was used for statistical analysis.

**Results:**

Three observational studies met our inclusion criteria. The reported prevalence of SIADH in the constituent studies ranged from 3 to 56%. The quantitative synthesis, encompassing 306 patients’ data, revealed a pooled SIADH prevalence of 20% (95% CI 0.00–52%, I^2^ 96%). The quality assessment revealed a moderate quality of included studies. The results were heterogeneous, as depicted by a high I^2^.

**Discussion and conclusion:**

The results from this review revealed a relatively high prevalence of SIADH of 20% in patients with brucellosis. Thus, hyponatremia in patients with chronic fever should prompt SIADH and brucellosis workup, particularly in endemic brucellosis areas. Likewise, patients with brucellosis merit hyponatremia screening. More extensive studies are needed to ascertain the exact prevalence of hyponatremia and SIADH in this patient cohort and their impact on the diagnosis and the overall prognosis.

## 1 Introduction

Hyponatremia is a state of excess water relative to sodium concentration present in the extracellular compartment (serum sodium, <135 mmol per liter). It can be associated with significant morbidity if not identified and appropriately treated and occasionally can be the first indicator of underlying diseases [1,2].

The syndrome of inappropriate secretion of antidiuretic hormone (SIADH) is a common cause of hyponatremia. It is usually a secondary disorder caused by certain drugs, pulmonic diseases, infections, central nervous system diseases, malignancy, amongst other causes [1,3]. These disorders trigger an unregulated vasopressin release. The dysregulated vasopressin release leads to a euvolemic state with low serum osmolality and sodium levels with increased urine sodium and osmolality [1,3].

Brucellosis is an infectious zoonotic disease caused by gram-negative coccobacillus. It affects humans and animals [4,5]. It is transmitted via ingestion of sheep, goat, and cattle products or close contact with their infected tissues. It is one of the most common bacterial zoonosis globally, yet it remains largely underdiagnosed and under-reported [4,6,7]. This is likely due to itschronic tendency, its non-specific clinical features, the slow rate of brucella growth in blood cultures, and suboptimal serological testing. Notwhistandingthis, brucellosis can significantly risk disability and morbidity; hence, timely diagnosis and management are crucial [4].

We previously reported a brucellosis case manifesting as reversible ataxia induced by SIADH-associated hyponatremia [8]. Visiting the literature revealed very scarce data about the relationship between SIADH and brucellosis [11,^9^,10]. Thus, in this report, we attempted to systematically review the literature and examine the prevalence of SIADH in patients with brucellosis.

## 2 Methods

A systematic review and a proportion meta-analysis keeping with PRISMA guidance [12]. The preliminary results of this review were published as an abstract at the Endocrine Society conference (ENDO 21) [13].

### 2.1 Study eligibility criteria

We included observational studies that examined the prevalence of SIADH in brucellosis patients. The included study had to have confirmation of brucellosis and SIADH.

### 2.2 Search strategy

We conducted a comprehensive search of PubMed, Medline, EMBASE, and Google Scholar since their inception through 30/04/2021. We did not have date or language limitations.Example of a database search strategy is: (((((((siadh[MeSH Terms]) OR (syndrome of inappropriate adh siadh secretion[MeSH Terms])) OR (adh syndrome, inappropriate[MeSH Terms])) OR (antidiuretic hormone, inappropriate secretion[MeSH Terms])) OR (SIADH)) OR (antidiuretic hormone)) OR (inappropriate vasopressin)) AND (((((((((Brucellosis) OR (Brucella)) OR (Brucella infection)) OR (Cyprus Fever)) OR (Maltese Fever)) OR (Undulant Fever)) OR (brucella[MeSH Terms])) OR (brucelloses[MeSH Terms])) OR (brucellosis[MeSH Terms])) (Supplementary 1). Furthermore, we performed a manual reference search and free text search to add to the review comprehensiveness.

### 2.3 Screening and data extraction

Title and abstract screening were conducted by two reviewers (MFHM & AOEA). Potentially eligible articles were imported for full-text assessment for possible inclusion. A third reviewer (HM) settled discrepancies following the protocol whenever a disagreement was not solved by discussion. We extracted data using an excel sheet. Data collected included the first author, year of publication, study type, the prevalence of SIADH, brucellosis diagnostic criteria used in the specific study.

## 3 Outcome

The primary outcome was the proportion of SIADH in brucellosis patients.

### 3.1 Study quality and risk of bias assessment

We used the modified hoy et al. tool used to assess the quality of the included prevalence studies [14]. We additionally generated DOI and funnel plots to examine the publication bias risk.

### 3.2 Data analysis

We used a proportion meta-analysis with double arcsine transformation and back transformation to stabilize the variance and confine the confidence interval between 0 and 1 [15]. We generated forest plots to display the analysis results. We used I squared (I2) to adjudicate the heterogeneity. We considered an I^2^ >60% to be indicative of significant heterogeneity. Regardless of the heterogeneity level, we decided to use the random-effects model (REM) in our analysis. MetaXl software was utilized for statistical analysis (version 5.3 © EpiGear International Pty Ltd ABN 51 134 897 411 Sunrise Beach, Queensland, Australia, 2011–2016).

## 4 Results

**Fig. 1:**
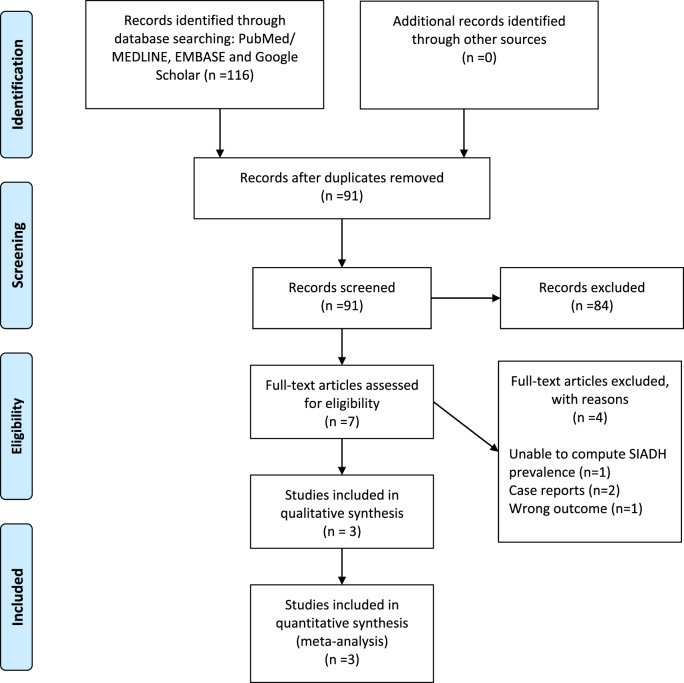
Flow diagram.

The broad initial database search has retrieved 116 potentially relevant articles. Ninety-one articles remained after duplicate removal, of which seven articles were assessed in full for eligibility. Finally, three articles encompassing 306 patients were included in our quantitative synthesis (Fig. 1). The oldest study that attempted to examine the prevalence of SIADH in brucellosis patients by Aysha et al. was excluded from our review as the SIADH prevalence could not be ascertained accurately [9].

### 4.1 Included studies baseline characteristics

The first included study was in Turkey (2014) by Dulger et al., who studied 35 adult brucellosis patients prospectively and found 54% (19 patients) prevalence of SIADH-associated hyponatremia [10]. The second study was also in Turkey by Bala et al. (2016) and studied the frequency of SIADH in children and adolescents with SIADH (0–18 years) [11]. Bala et al. reported a prevalence of SIADH of 21.9% in this population. The third study was conducted in China by Zhao et al., in 2019, who studied SIADH prevalence in 111 brucellosis patients and found it to be 3% using laboratory criteria [16]. In their study, Zhao et al. reported hyponatremia prevalence of 10% (11/111), and as not all these patients had SIADH screening, they reported another frequency of SIADH based on clinical suspicion. They reported it to be 9% (10/111) (Table 1 summarizes the included studies’ baseline characteristics).

**Table 1:** Summary of included studies baseline characteristics.

Study	Total number of patients involved in the study	Mean or median age (Male %)	Brucellosis Diagnosis	SIADH Criteria	Cases of SIADH/population n (%)	Average serum sodium level	Average urine sodium level	Average serum osmolality	Average urine osmolality	Remarks
**Zhao 2019 (China)** [16]	111 (adult patients)	55 years	Positive brucella titer or blood culture	Serum NA^+^ <135 mmol/L, osm <275 mosm/L. Urine Osm >100 mosm/L and Urine NA^+^ > 30 mmol/l Absence of kidney, thyroid , adrenal disease or diuretic use.	3/111 (2.7% confirmed cases) 10/111 (9% suspected cases)	119 mmol/L	193 mmol/l	272 mosm/L	547 mosm/L	The details provided in the table are of the three confirmed cases only. The details of Non-SIADH patients were not provided.
**Bala 2016 (Turkey)** [11]	160 (children and adolescents).	9.58 years (56.2%).	Positive brucella titer >1:160 in standard Wright tube agglutination tests or positive culture.	Serum Na+ <135 mmol/L+ osm <275 mosm/L. Urine NA^+^ > 25 mmol/l with with normal dietary salt intake, low uric acid (<2 mg/dL). Clinical euvolemia and absence of kidney, thyroid, adrenal disease or diuretic use.	35/160 (21.9%).	In SIADH group = 130.8 (±3.4) Non-SIADH group = 141.4 (±25.9)	NS	274.6 (±5.50) mosm/lt	239.4 (±90.32) mosm/kg	Significant association between SIADH higher serum glucose, ALT, AST, LDH, CRP and lower albumin, K^+^ and Hb.
**Dulger 2014 (Turkey)** [10]	35 (adult patients)	38 years (71%) male	Positive brucella titer >1:320 or positive culture.	Serum Na+ <135 mmol/L+ osm <275 mosm/L. Urine NA^+^ > 40 mmol/l with normal dietary salt intake. Clinical euvolemia, and absence of renal, thyroid, and adrenal disease, or recent use of diuretic agents.	19/35 (54%)	NS	132 mmol/L (range 40–224).	NS	NS	Significant association between SIADH and lower serum albumin and higher serum globulin levels.

NA^+^ = Sodium; Osm = Osmolality; NS = not specified; ALT = alanine aminotransferase; AST = aspartate aminotransferase; LDH = lactate dehydrogenase; CRP= C-reactive protein; Hb = hemoglobin; K^+^=Potassium.

### 4.2 The prevalence of SIADH in brucellosis patients

**Fig. 2:**
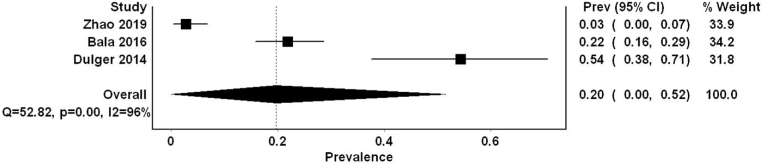
Forest plot summarizing the proportion of SIADH in patients with brucellosis.

Our analysis revealed a 20% prevalence of SIADH in brucellosis patients (proportion 0.2, 95% CI 0.0–0.52) (Fig. 2). The results were markedly heterogeneous, as depicted by an I^2^ of 96%. Using the clinical criteria of SIADH in the study by Zhao et al. led to an increase in the overall prevalence of SIADH in brucellosis patients to 24% (proportion 0.24, 95% CI 0.04–0.48, I^2^ 93%) (supplementary 2) [16].

### 4.3 Sensitivity and subgroup analyses

Sensitivity analysis revealed that the exclusion of the Zhao et al. study would lead to an increase in the overall prevalence to 35% (proportion 0.35, 95% CI 0.05–0.71, I^2^ 92%) [16]. The exclusion of Dulger et al.’s study, which has the highest report SIADH prevalence in brucellosis patients, would drop the pooled prevalence to 10% (proportion 0.10, 95% CI 0.00–0.34, I^2^ 96%) (Supplementary 3) [10]. The scarcity of data prohibited any meaningful subgroup analysis.

### 4.4 Risk of bias assessment

The quality of included studies was mainly moderate based on the modified Hoy et al. tool (Table 2). The DOI plot showed no asymmetry, suggesting publication bias's absence (Supplementary 4). For comparison, a Funnel plot was populated, and it depicted a marked asymmetry, suggesting the possibility of publication bias.

**Table 2:** Table summarizing the risk of bias assessment.

Study	1	2	3	4	5	6	7	8	9
**Zhao 2019** [16]									
**Bala 2016** [11]									
**Dulger 2014** [10]									

low risk; high risk;  unclear risk assessment.

(1) Was the study's target population a close representation of the national population in relation to relevant variables, e.g. age, sex, occupation?; (2) Was the sampling frame a true or close representation of the target population?; (3) Was some form of random selection used to select the sample, OR, was a census undertaken; (4) Was the likelihood of non-response bias minimal?; (5) Were data collected directly from the subjects (as opposed to a proxy); (6) Was an acceptable case definition used in the study?; (7) Was the study instrument that measured the parameter of interest (e.g. prevalence of low back pain) shown to have reliability and validity (if necessary)?; (8) Was the same mode of data collection used for all subjects?; (9) Were the numerator(s) and denominator r(s) for the parameter of interest appropriate.

## 5 Discussion

In this first review examining the relationship between SIADH and brucellosis, we found a relatively high pooled prevalence in brucellosis patients (20%, 95% CI 0–52%). This finding may help clinicians suspect brucellosis in patients presenting with chronic fevers, non-specific complaints in the presence of SIADH, and hyponatremia. Furthermore, it will form the base for future related prospective studies that assess the prevalence and prognostic value of SIADH in this patient cohort.

The mechanism of SIADH in brucellosis and other infectious diseases is not well elucidated. Direct involvement of the central nervous system can lead to ectopic ADH secretion and SIADH [17]. Decreased venous return, hypoxemia, and hypercapnia that occasionally accompany lung infections are proposed to induce a non-osmotic ADH release [[18], [19], [20], [21], [22]]. Regulation of serum sodium levels assuming at newer lower target (e.g., 125–135 mmol/L), also known as reset osmostat, was described in malaria and tuberculosis cases [23,24]. Proinflammatory cytokines, namely interleukin-6, can lead to ADH stimulation [25,26]. Lastly, stress, nausea, and vomiting that accompany infections can also lead to non-osmotic ADH secretion [27].

In Dulger et al.’s study, SIADH was found to be significantly associated with lower serum albumin and higher globulins. Increased urine sodium was found to be correlated with these. Dulger hypothesized the increased severity of brucellosis associated with increased urinary sodium secretion and hypothesized a possible future diagnostic role for SIADH in brucellosis cases [10]. Limitations to Dulger's study were excluding neuro-brucellosis cases, which may have affected the exact overall SIADH prevalence. Additionally, there was no uniform urine screening with osmolarity measurements as a part of the SIADH diagnostic criteria.

Bala et al.’s study were the largest (160 patients) amongst our constituent studies [11]. In this study, SIADH was found to be associated with higher serum blood glucose (p < 0.001), alanine aminotransferase (ALT) (p < 0.05), aspartate aminotransferase (AST) (p < 0.05), lactate dehydrogenase (LDH) (p < 0.001), C-reactive protein (CRP) (p < 0.001); and reduced albumin (p < 0.001), total protein (p < 0.05), potassium (p < 0.05), and hemoglobin (p < 0.05). The findings that higher CRP, ALT, AST, and lower albumin were seen in SIADH patients prompted the authors to suggest an association between SIADH and severe inflammation or disease. It is noteworthy that SIADH in this study responded dramatically to brucellosis treatment and fluid restriction [11].

Our review has limitations such as the inclusion of a limited number of studies with a small number of included studies, the heterogeneity of the study results, the likely possibility of publication bias either due to overlooking or underreporting this association, and varying age included patients in the constituent studies. Nonetheless, we included all available studies in this first attempt to review this association systematically, and our review yielded important results.

In conclusion, this first review and meta-analysis explore the relationship between SIADH and brucellosis. The findings from this synthesis and its constituent studies suggest a high prevalence of SIADH in brucellosis patients, additionally, the possibility of SIADH correlating with severe disease and inflammation. Additionally, it highlighted the possible utility of SIADH in future brucellosis diagnostic and perhaps prognostic tools. Moreover, it hinted at the importance of screening for hyponatremia in brucellosis patients. More extensive prospective studies will further add to the results of this synthesis.

## Ethical approval

Ethical approval is not required for this analysis as it is a secondary synthesis of publicly available data.

## Author’s contribution

Conceptualization: MFHM; Supervision: MFHM; Writing initial draft: MFHM; AOEA, MBH, AOS, YSA. Editing the first draft: MFHM, ZASM, LA and HFHM. Article's screening: MFHM and AOEA; Data extraction: AOEA, MBH, AOS, and MFHM; Statistical analysis: MFHM; Tables and diagrams: AOEA, MBH, AOS, and MFHM. Review and editing: All authors; Approval of the final manuscript: All authors.

## Consent

N/A

## Registration of Research Studies

Name of the registry: The Prevalence of Syndrome of Inappropriate Antidiuretic Hormone Secretion (SIADH) in Brucellosis Patients: A Protocol for a Systematic Review and a Meta-analysis

Unique Identifying number or registration ID: PROSPERO 2020 CRD42020176730

Hyperlink to your specific registration (must be publicly accessible and will be checked): https://www.crd.york.ac.uk/prospero/display_record.php?ID=CRD42020176730.

## Data availability statement

The data generated from the analysis are presented in this article. If any other specific data is needed, a request can be made to the corresponding author, who will make sure these are available upon reasonable request.

## Funding

None.

## Declaration of competing interest

The authors disclose no conflict of interest related to the writing, analysis, or publication of this paper.
