# Supplementary material for: The prevalence of the syndrome of inappropriate antidiuretic hormone secretion (SIADH) in brucellosis patients: Systematic review and meta-analysis
Source: Ann Med Surg (Lond). 2022 Feb 5;74:103340. doi: 10.1016/j.amsu.2022.103340 (PMC8844796; doi:10.1016/j.amsu.2022.103340)
Supplement: Multimedia component 1 [file mmc1.docx]

**Supplementary 1:** Table showing the search queries used in this review.

| EMBASE search query | PubMED Search query |
| --- | --- |
| (brucellosis OR 'brucellosis epidemiology' OR 'brucellosis'/exp OR brucell*) AND ('inappropriate vasopressin secretion' OR 'inappropriate vasopressin secretion'/exp OR 'syndrome of inappropriate antidiuretic hormone secretion' OR siadh) | (((((((siadh[MeSH Terms]) OR (syndrome of inappropriate adh siadh secretion[MeSH Terms])) OR (adh syndrome, inappropriate[MeSH Terms])) OR (antidiuretic hormone, inappropriate secretion[MeSH Terms])) OR (SIADH)) OR (antidiuretic hormone)) OR (inappropriate vasopressin)) AND (((((((((Brucellosis) OR (Brucella)) OR (Brucella infection)) OR (Cyprus Fever)) OR (Maltese Fever)) OR (Undulant Fever)) OR (brucella[MeSH Terms])) OR (brucelloses[MeSH Terms])) OR (brucellosis[MeSH Terms])) |

**Supplementary 2:** Forest plot after adding suspected SIADH cases in Zhao study to the analysis.


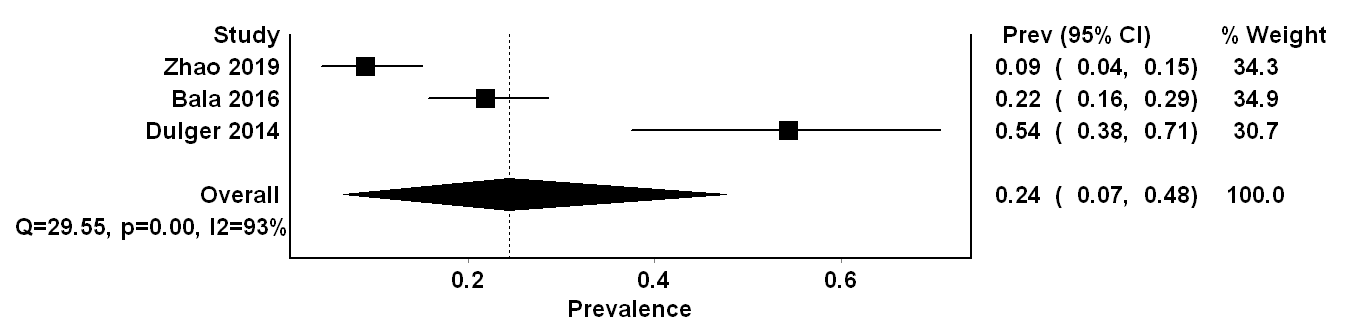


**Supplementary 3:** Table showing the results of sensitivity analysis

| **Excluded study** | **Pooled Prevalence** | **LCI 95%** | **HCI 95%** | **Cochran Q** | **p** | **I 2** | **I 2 LCI 95%** | **I 2 HCI 95%** |
| --- | --- | --- | --- | --- | --- | --- | --- | --- |
| **Zhao 2019** | 0.351668579 | 0.058962564 | 0.711153041 | 13.31064062 | 0.000263904 | 92.48721359 | 74.56010805 | 97.78136009 |
| **Bala 2016** | 0.228590992 | 0 | 1 | 45.48753253 | 1.54E-11 | 97.80159542 | 94.71936851 | 99.08477183 |
| **Dulger 2014** | 0.107548543 | 0 | 0.342790967 | 25.54133353 | 4.33E-07 | 96.08477765 | 88.99883108 | 98.6066057 |

**Supplementary 4:** Figure 1 showing DOI plot showing absence of asymmetry suggestive of the absence of a publication bias


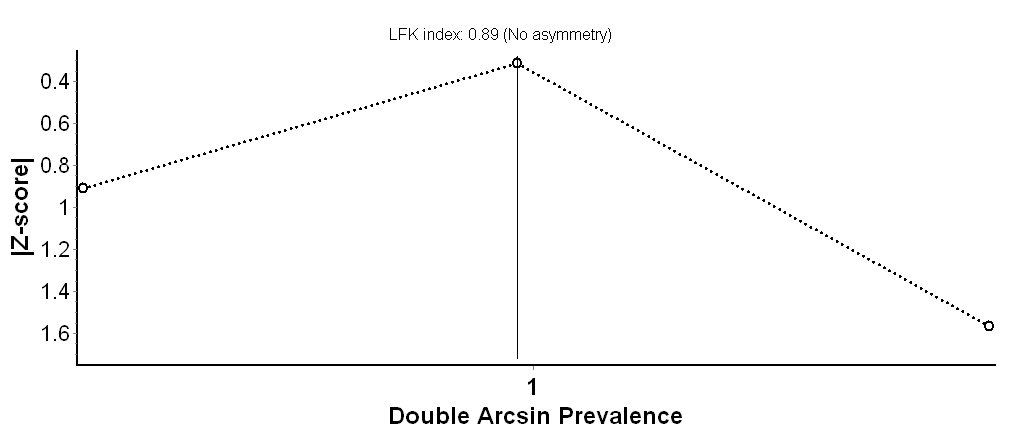


*The small number of studies in this analysis may have limited proper assessment of publication bias.
